# Advances in the superhydrophilicity-modified titanium surfaces with antibacterial and pro-osteogenesis properties: A review

**DOI:** 10.3389/fbioe.2022.1000401

**Published:** 2022-09-06

**Authors:** Hanyu Shao, Mingchen Ma, Qiang Wang, Tingting Yan, Baohong Zhao, Shu Guo, Shuang Tong

**Affiliations:** ^1^ Department of Plastic Surgery, First Hospital of China Medical University, Shenyang, China; ^2^ School and Hospital of Stomatology, China Medical University, Shenyang, China; ^3^ Faculty of Materials Science and Engineering, Kunming University of Science and Technology, Kunming, China

**Keywords:** titanium, superhydrophilicity, UV photo-functionalization, plasma treatment, antibacterial, osseointegration, biocompatibility

## Abstract

In recent years, the rate of implant failure has been increasing. Microbial infection was the primary cause, and the main stages included bacterial adhesion, biofilm formation, and severe inhibition of implant osseointegration. Various biomaterials and their preparation methods have emerged to produce specific implants with antimicrobial or bactericidal properties to reduce implant infection caused by bacterial adhesion and effectively promote bone and implant integration. In this study, we reviewed the research progress of bone integration promotion and antibacterial action of superhydrophilic surfaces based on titanium alloys. First, the adverse reactions caused by bacterial adhesion to the implant surface, including infection and bone integration deficiency, are briefly introduced. Several commonly used antibacterial methods of titanium alloys are introduced. Secondly, we discuss the antibacterial properties of superhydrophilic surfaces based on ultraviolet photo-functionalization and plasma treatment, in contrast to the antibacterial principle of superhydrophobic surface morphology. Thirdly, the osteogenic effects of superhydrophilic surfaces are described, according to the processes of osseointegration: osteogenic immunity, angiogenesis, and osteogenic related cells. Finally, we discuss the challenges and prospects for the development of this superhydrophilic surface in clinical applications, as well as the prominent strategies and directions for future research.

## 1 Introduction

Implants are used in orthopedic, dental care, and cardiovascular devices. The most commonly used metallic materials for implants are stainless steel Arciola et al. (2018), titanium, and titanium alloys. Metal implants have been used in biomedicine since the 19th century. They are used as temporary and permanent implants in the body. Several properties of metals are suitable for bone repair. The tensile strength of metals is greater than that of polymers, their toughness is 20 times higher than that of ceramics, and their fatigue life is reasonable. Metals can be alloyed, thereby making them resistant to corrosion. In addition, using elements that do not adversely affect the body can improve biocompatibility. Thus, metals have been used for implantable device applications, and this trend is not expected to change soon. Titanium was developed for aerospace applications in the 1950’s; however, after the 1960’s, it was used in surgical implants. Titanium has gained popularity because of its excellent combination of strength, Young’s modulus, and biocompatibility compared with other metal implant materials (Kaur and Singh, 2019). For all their advantages, metal implants pose potential risks to bacterial infection, either from the patient’s body or the surgical instruments in the operating room (Jaggessar et al., 2017). Bacteria easily adheres to the implant materials, and microbial infection accelerates the corrosion and loosening of the implant as well as increases the probability of repeated surgery. For example, joint replacement prosthetic infections occur in approximately 1% of joint replacements, a proportion that increases significantly during revision surgery (Campoccia et al., 2006; Moran et al., 2010). In dentistry, clinical studies at five years of follow-up have shown that up to 14.4% of implants are surrounded by implant infections, with the incidence increasing over time (Norowski and Bumgardner, 2009). Microbial infection implants can lead to prolonged hospitalization and increase bacterial drug resistance while contributing to the evolution of superbugs, which can lead to death or amputation in severe cases. It may also turn into a chronic infection (Ferraris and Spriano, 2016).

When bacteria adhere to the implant, periprosthetic biofilm layers predominantly consisting of proteins and polysaccharides that are highly resistant to antimicrobial therapy are formed, which can lead to local infections or even deeper and more serious systematic infections (Jaggessar et al., 2017). Timely bone integration plays a critical role in the occurrence of bacterial adhesion. If bacterial adhesion occurs before tissue repair, host defense cannot prevent surface colonization and biofilm formation (Arciola et al., 2018), and bacterial adhesion during the first few hours of cell contact with the biomaterial may impair the entire process of bone healing; that is, processes such as cell adhesion, cell differentiation, and subsequent nutrition are impaired by bacterial colony formation on the implant surface. In addition, local inflammatory processes may be established, leading to changes in tissue pH and the migration of defense cells, such as macrophages, fibroblasts, and white blood cells, consequently affecting bone healing (Kunrath et al., 2020). Osseointegration results from inflammatory driving processes on and around the implant surface. A favorable immune response can promote osseointegration and wound repair, whereas an undesirable immune response can lead to excessive inflammation, pain, tissue destruction, fiber wrapping, and even implant failure (Anderson et al., 2008). To avoid the adverse effects of bacterial adhesion, scientists have also proposed several methods, and the main modes of action of commonly used antibacterial agents can be summarized as follows (Zhan et al., 2021): 1) Destroying or inhibiting the cell wall synthesis process; 2) Inhibiting the function of the cell membrane; 3) Inhibiting the protein synthesis process of bacterial cells; 4) Combining with components of DNA or RNA synthesis process to inhibit nucleic acid synthesis and affect the normal reproduction process of bacteria; 5) Inhibiting other metabolic processes, such as the destruction of folic acid, which is important for DNA synthesis.

The ideal implant material should have antibacterial properties as well as favorable biocompatibility. In recent years, studies have increasingly been conducted on superhydrophilic surfaces. Among numerous antibacterial methods, superhydrophilic surfaces exhibit antibacterial effects while achieving excellent biocompatibility. It was found that osteoblasts proliferated faster, and they were larger, longer, and more active on superhydrophilic Ti surfaces (Henningsen et al., 2018). More filamentous extension of macrophages was also observed on the surface of anodized and hydrogenated titanium (with superhydrophilic properties), and the stretched appearance of these macrophages was significantly less after 24 h (Gao et al., 2020). Compared with the micro-smoothness of nano-titanium, nano-rough particles, nanotubes, and nano-braided titanium can enhance the adhesion of osteoblasts and also provides other functionalities, such as alkaline phosphatase synthesis, calcium deposition, and collagen secretion (Puckett et al., 2010; Wennerberg et al., 2014). As the superhydrophilic surfaces are rough, well-organized topography at nano/microscales could improve bio-compatibility and promote bone formation, which is crucial for successful osseointegration between the implant and bone. The cell filopodia can enter the pore of nanotubes to form a locked-in cell structure for bone ingrowth (Zhao et al., 2020). The hydrophilicity of implants has been identified as an important factor that may affect the early bone response, i.e., high hydrophilicity, faster healing, and therefore superior stability and the possibility of early loading, with favorable clinical predictability (Rupp et al., 2004). High hydrophilicity can improve the biological activity of biomaterials and promote cell adhesion (Li et al., 2019). Table 1 summarizes several studies on superhydrophilicity surfaces that promote osseointegration and the cells they affect. The antibacterial mechanisms and optimal biocompatibility of superhydrophilic surfaces were discussed, mainly with regard to anti-inflammatory properties and osteogenesis promotion.

**TABLE 1:** Example of superhydrophilic surfaces promoting osseointegration.

Type of alloy	Preparation methods	CA	Cell culture	Mechanism of action	Reference
Tantalum	Electrochemical anodization	0°	MC3T3-E1	Triggering FAK and YAP\/RUNX2 cell signaling pathways	Zhang Z. et al. (2021)
Si-TiO_2_	*In situ* anodization and Si plasma immersion ion implantation (PIII)	11.25 ± 0.88°	MC3T3-E1	The expression of Runx2 and ALP increased on Si-TiO_2_-NTs	Zhao et al. (2020)
Ti	Acid etching Thermal alkali	<10°	RAW 264.7	Inhibition of osteoclast related markers, most osteoclasts growing on the surface of the material were mononuclear	Kartikasari et al. (2022)
Ti6Al4V	Electrochemical anodization	5°	Osteoblast MG63 cells	The MTT results exhibited high cell viabilities of 98.1%	Rahnamaee et al. (2020)
Hydrogenated titanium dioxide (H_2_-TNT)	Electrochemical anodization Hydrogenation	3.65 ± 0.52°	Macrophages	H_2_-TNT surface elicited up-regulated gene expression of M2 surface markers and down-regulation of M1 surface markers	Gao et al. (2020)

## 2 Superhydrophilicity principle

Superhydrophilic structures are usually characterized by a contact angle (CA) less than 10°. CA is the reaction of surface wettability, and surface roughness and surface energy together determine wettability (Si et al., 2018). The surface energy calculated from the CA data shows that increasing the surface roughness increases the surface energy and at the same time increases the surface wettability, making the material superhydrophilicity (Puckett et al., 2010). More importantly, surface roughness and surface energy are key to favorable biocompatibility, and osteoblasts are more inclined to adhere to surfaces with high roughness and surface energy (Salido et al., 2007; Puckett et al., 2010).

Superhydrophilic materials were first inspired in 1970 *via* research on the human cornea, in which tears can completely diffuse across the cornea, forming a water membrane to eliminate the scattering of light (Si et al., 2018). In 2010, Zheng et al. (2010) reported that spider silk can efficiently collect water from the air, with the surface energy gradient and Laplacian pressure difference, generated by spider silk with a spindle structure, allowing continuous directional water condensation around the spider silk. The superhydrophilicity of the pitcher plant was discovered in 2016 (Chen et al., 2016), opening a new pathway for the study of superhydrophilicity structures. Currently, scientists have established a few relatively mature manufacturing methods for superhydrophilic materials, and they can be roughly divided into two categories: physical and chemical methods. Physical methods include laser treatment, physical vapor deposition, and spraying. Vorobyev and Guo created a novel method for achieving regular superhydrophilicity of silicon using high-intensity femtosecond laser pulses (Vorobyev and Guo, 2010). Because of its superhydrophilicity, water resists gravity by spreading vertically upwards. The driving force of water motion is the surface energy generated by the surface structure and the Laplacian pressure (Vorobyev and Guo, 2010; Si et al., 2018). Zheng et al. (2016) reported spray-dried superhydrophilicity TiO_2_/SiO_2_ nanoparticle coatings. Numerous procedures can be used to form a superhydrophilicity surface, on which a drop of water or blood will immediately spread and wet the surface. To achieve superhydrophilic surfaces, plasma treatment and ultraviolet (UV) irradiation are commonly used (Albrektsson and Wennerberg, 2019). Li et al. (2008) prepared a long-term stable superhydrophilicity-layered TiO_2_ via pulse laser deposition technology and annealing. The TiO_2_-layered particle array exhibits superhydrophilicity, with the water CA approaching 0°, without requiring further UV exposure. Chemical methods, such as a novel underwater superhydrophilic polyacrylamide hydrogel coated mesh, are relatively complex, and can be used to achieve the selective separation of oil and water mixtures with high separation rates (Si et al., 2018).

## 3 Effect of superhydrophilicity surface on bacteria

### 3.1 Bacterial adhesion

**FIGURE 1:**
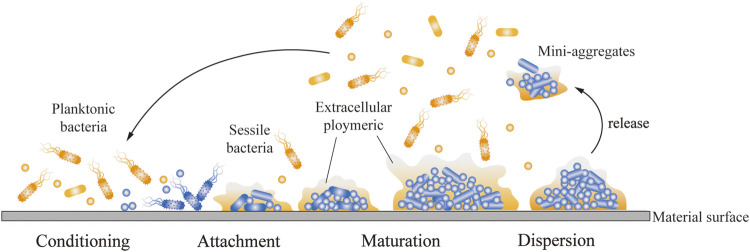
Process of bacterial adhesion on implant surface. The planktonic bacteria attach and adhere to the implant surface and bind to the sessile bacteria. The bacteria covered by EPS gradually mature and continue secreting EPS to attract more bacteria to adhere and form biofilms. An aggregate of bacteria began to break up into mini-aggregates, which were released.

As shown in Figure 1, when bacteria stick to the implant surface, the human body shows inflammation in response to foreign metals and pathogens. The inflammatory response of the host contributes to the formation of biofilms, because molecules produced as part of this response help the bacteria adhere to the surface of the medical device (Lin and Bumgardner, 2004). Therefore, inflammation can cause implant trauma and damage to the underlying bone. The implantation of infected bacteria is generally not a sparse distribution of single adherent cells, but rather a biofilm in which bacterial aggregates adhere tightly to the surface of the biomaterial and are encased in a large matrix of extracellular polymers (EPSs) (Arciola et al., 2018). The growth of biofilms, harsh physical environment, and sublethal concentrations of antibiotics can serve as stress signals to stimulate persistent cell formation, which is responsible for the persistence of implant infections and the source of the spread of bacteria to other parts of the body. In addition, chronic inflammation occurs, because host immune defenses and traditional antimicrobial therapies are often ineffective against bacteria growth in biofilms. Different microbial species, including Gram-negative bacteria, Gram-positive bacteria, and fungi can form biofilm to against adverse factors. In addition, the high cell density in biofilms alters microbial gene expression, contributes to their increased virulence, and enhances inter-bacterial adhesion, consequently resulting in more frequent binding between biofilm community members than that between planktonic bacteria (Arciola et al., 2018).

### 3.2 Antibacterial mechanism of superhydrophilic surface

A thin oxide layer, TiO_2_, was formed on the titanium surface when titanium was exposed to air, and the oxide layer surface generally absorbed organic hydrocarbon contaminants from the atmosphere (Zhao et al., 2007; Att et al., 2009). Surface modification technologies can diminish hydrocarbon contamination, increase the content of functional OH groups on the material surface, and endow titanium with superhydrophilicity without altering the surface topography (Choi et al., 2016; Matsumoto et al., 2020). Furthermore, treatments, such as UV irradiation and plasma treatment, can directly inactivate bacteria and biofilms on the titanium surface while obtaining superhydrophilicity (Koban et al., 2011; Guo et al., 2021), thereby creating a sterile environment for implantation. However, resistance to bacterial adhesion during or after implantation is also an aspect that cannot be ignored. Bacterial adhesion is a complex physical and chemical process that includes three stages: transport of bacteria towards a surface, reversible bacterial adhesion, and transition from reversible to irreversible adhesion (Yang et al., 2022). A 6 h post-implantation period has been identified as a “decisive period,” during which the inhibition of bacterial adhesion is critical to the long-term success of an implant (Zilberman and Elsner, 2008). Thus, the antibacterial effects on the first day are crucial to ensuring successful implantation.

The effect of surface wettability, that is, whether the material is hydrophilic or hydrophobic, on bacteria adhesion is currently inconclusive. Studies have indicated that hydrophobic surfaces could reduce the velocity of bacteria through collisions and slightly increase the collision duration when bacteria approach the surface, thereby promoting the landing and adhesion of bacteria (Qi et al., 2017), whereas hydrophilic surfaces could form more hydrogen bonds with bacteria, leading to increased adhesive forces, even exceeding the adhesion force on the hydrophobic surface (Boks et al., 2008).

The mechanisms of bacterial adhesion on the surface of materials are complex; they are related to the characteristics of the material itself, such as surface patterning, roughness, wettability, and surface charge as well as the characteristics of different bacteria (Pajerski et al., 2020; Yang et al., 2022). Without considering the addition of antibacterial ingredients or changing surface morphology, superhydrophilic surfaces can be obtained with certain bacteriostatic properties (Lan et al., 2015; Jeong et al., 2017). Bacteria were generally not completely eliminated on the titanium surface, so the reduction in the number of bacteria was attributed to the anti-adhesion effect, and the bacterial viability was inhibited by the treatment on the superhydrophilic titanium surface. This type of superhydrophilic surface with short-term antibacterial effects can meet the requirements of clinical implantation without infection, and its antibacterial mechanisms are largely dependent on surface treatment methods. Therefore, we selected the typical surface modification methods, UV photo-functionalization and plasma treatment, and their several potential bacteriostatic mechanisms. Table 2 lists several examples of superhydrophilic surfaces with antibacterial properties.

**TABLE 2:** Examples of bacteriostasis on superhydrophilic surfaces.

Material	Processing mode		Bacterial	Experimental results	Reference
	Equipment	Processing time			
Ti	UV light	12 min	*S. aureus*	Higher antibacterial activity with increased culture time, and enhance the phagocytic ability of macrophages	Yang et al. (2021)
Ti	PECVD system (F and O dual plasma-base)	—	*S. aureus*	Antibacterial rates: fresh F-O-Ti 100%, after 1 day 96.6%, after 3 days 90.5%, after 7 days 89.8%	Chen et al. (2019)
Ti/Ti-Ag	Non-thermal atmospheric pressure plasma jet (NTAPPJ)	10 s	*S. sanguinis*	Bacterial adhesion was significantly reduced, the change of ions had no obvious effect on bacterial adhesion resistance	Lee et al. (2017)
Ti	NTAPPJ	10 min	*S. sanguinis*	The structure of aggregates changed from a long-chain shape to a short-chain form	Jeong et al. (2017)
Ti/Ti6Al4V	UV(λ = 254 nm, 8–10 mW/cm^2^)	15 min	*S. aureus*	The antimicrobial activity was maintained for seven days after UV irradiation	Itabashi et al. (2017)
Ti	UV (λ = 254 nm, 100 mW/cm^2^)	15 min	*Actinomyces oris*	During the initial attachment period, *Actinomyces oris* colonization is reduced and biofilm formation is inhibited for up to 6 h	Zhang et al. (2017)
Ti	NTAPPJ	2–10 min	*S. mutans*	Both adhesion and the biofilm formation rate were significantly lower for Gram-negative bacteria than Gram-positive bacteria on samples treated for longer durations with the NTAPPJ	Lee et al. (2019)
			*S. aureus*		
			*Klebsiella oxytoca*		
			*K. pneumoniae*		
Ti	UV (*λ* = 365 nm, 10 mW/cm^2^)	5 min	*P. aeruginosa*	After 30 min, *P. aeruginosa* decreased by 90%, but by 240 min, *S. aureus* reduced by more than 99%	Pan et al. (2021)
			*S. aureus*		

#### 3.2.1 Ultraviolet photo-functionalization

The naturally occurring oxide film on the titanium surface generally exists in an amorphous state and does not exhibit photocatalytic ability; however, the three crystal structures of TiO_2_, anatase, rutile, and brookite, show photocatalytic activity and can be obtained via various oxidation methods, including sol-gel method, sputtering, chemical vapor deposition, atomic layer deposition, plasma immersion ion implantation, cathodic arc deposition, and anodization (Yeniyol et al., 2015; He et al., 2019). Under the excitation of UV light, the TiO_2_ surface with semiconductor properties can generate electron-holes pairs, inducing a series of photocatalytic reactions, and facilitating the antibacterial effect (Chouirfa et al., 2019). UV treatment on titanium surfaces leads to the excitement of electrons from the valence band to the conduction band, followed by the abundant production of electron-hole pairs. Gallardo-Moreno et al. (2010), reported that the irradiation of Ti6Al4V surfaces with UV-C light produced residual post-radiation effects that directly affected the viability of adhered bacteria, and the antibacterial effects are likely due to the return of the absorbed energy and the formation of little electrical currents caused by the surface charge during the relatively slow recombination process of electron-holes pairs of TiO_2_ after irradiation. Hatoko*et al.* reported that UV-treated titanium surface inhibited the proliferation of *S. aureus* owing to the increased intracellular reactive oxygen species (ROS) (Hatoko et al., 2019). ROS can damage bacterial membranes and cell walls; thus, additional to destroying the bacterial defense system, they can also penetrate bacterial membranes, and destroy proteins and lipids, directly or indirectly disrupting cellular respiration and other physiological activities (Ren et al., 2020). UV treatment renders the titanium surface with a bacteria repellent; however, the effect is time-dependent (de Avila et al., 2015; Zhang et al., 2017). Electrochemical anodization, a surface modification method, is often used to obtain a functional TiO_2_ film in the preparation of superhydrophilicity surfaces treated using UV. This anodized surface is inherently antibacterial. The surface of the titanium implant was placed in a sodium chloride solution and anodized by forming TiCl_3_ surface layer (Han et al., 2016). Subsequently, the modified surface gradually hydrolyzes, resulting in the formation of Ti–OH and bactericidal hypochlorous acid. Ti-OH endows the titanium surface with superhydrophilicity (Shibata et al., 2010), thereby facilitating favorable biocompatibility. Hypochlorous acid can be continuously released from titanium the surface for eight weeks (Shibata and Miyazaki, 2014), endowing the titanium surface with antibacterial properties (Shibata et al., 2010).

#### 3.2.2 Cold plasma treatment

Cold plasma, a neutral-ionized gas regarded as the fourth fundamental state of matter (other than solid, liquid, and gas) (Burm, 2012), is currently applied in the surface modification of materials. Plasma is a mixture that contains UV and heavy (molecules, atoms, free radicals, ions) and light (electrons and photons) species generated by the excitation of gas via electric discharges (Moreau et al., 2008). The oxide layer on the titanium surface can be modified using ions, such as COOH^−^, NO^−^, OH^−^, N^3−^, and O^2−^, after plasma treatment, and reactive oxygen and nitrogen species are the main effective components of cold plasma, enabling the titanium surface to perform reductive potential, which can oxidize the surrounding matter. Additionally, plasma-treated superhydrophilic titanium surfaces can exert bacteriostatic function through the ROS pathway (Yoo et al., 2015). Lee et al. (2019) reported that plasma-treated superhydrophilic titanium surfaces can inhibit the growth of Gram-negative bacteria; this inhibitory effect on Gram-negative bacteria is stronger than that on Gram-positive bacteria because of the thickness of the peptidoglycan layer in the bacterial cell wall (Lee et al., 2019). However, the contents of the reactive species in the materials were time-dependent, with the bacteriostatic effects decreasing over time (Park et al., 2018; Yang et al., 2021).

These two treatments show strong antimicrobial activity against Gram-negative bacteria, and the superhydrophilic surfaces also have a certain inhibitory effect on the formation of Gram-negative bacteria biofilm, such as the *P. aeruginosa* biofilm. After UV treatment of Ti plate, the growth of *P. aeruginosa* density and coverage decreased significantly, after 16 h of biofilm formation, UV treatment of titanium plate compared with untreated plate, the cumulative biomass significantly reduced and UV treatment on the surface of engraftment significantly sparser, cells less, smaller and more fragmented. The titanium discs were covered with larger, higher, and more extensive microcolonies (de Avila et al., 2015). Plasma nitriding Ti surface had excellent biofilm performance, and no large bacterial clusters of *P. aeruginosa* were observed after 3 or 6 h of culture, which may be related to the trivalent titanium ions produced by nitriding mechanism (Nunes Filho et al., 2018). Furthermore, the surface of Ti treated with non-thermal plasma (NTP) alone did not show the performance of effective inhibition of *P. aeruginosa* biofilm, but after the combination with gentamicin (GTM), the biofilm coverage area was significantly reduced. When treated with 0.25 h NTP and then 8.5 mg/L GTM, *P. aeruginosa* ATCC 15442 mature biofilm was completely eliminated from the surface. Therefore, NTP can be used as a suitable antibiofilm agent in combination with antibiotics for the treatment of biofilm-associated infections caused by this pathogen (Paldrychová et al., 2019; Paldrychova et al., 2020).

The chemical change, in which carbon content decreases and oxygen content increases on the surface of titanium after superhydrophilic modification (Hotchkiss et al., 2016; Jeong et al., 2017; Lee et al., 2017; Wang et al., 2020), can induce bacterial adhesion resistance. The effect of chemical composition on bacterial adhesion may be more important than surface energy, but further studies are needed to confirm this hypothesis (Lee et al., 2017). The bacteriostatic ability of the surface is optimal when the surface modification is completed, so in practical application, it is required to properly determine the ideal time point of surface treatment and implantation to better exert the bacteriostatic properties.

### 3.3 Comparison of antibacterial principle with superhydrophobic surface

Superhydrophobic surfaces have attracted extensive attention owing to their excellent self-cleaning and anti-fouling effects. If superhydrophobic surfaces are applied to implant surfaces, they can effectively reduce infection caused by microorganisms, reduce the rate of secondary surgery, as well as reduce thrombosis, thereby facilitating patient recovery (Kattula et al., 2017). Unlike superhydrophilicity surfaces, which are chemically antibacterial by changing some reactive oxygen groups or charges, superhydrophobic surfaces tend to directly kill bacteria that adhere to the surface. The antibacterial effect of superhydrophobic surfaces can be reflected in two aspects. On the one hand, superhydrophobic materials can prevent or reduce bone marrow-derived cells and bacterial adhesion (*S. aureus* and *verdigris*); this is owing to the reduction in the surface energy of the superhydrophobic surface and the amount of protein adsorption on the surface, thereby making the bacteria harder to adhere and more likely to be removed before the biofilm is generated, known as the self-cleaning effect of the superhydrophobic surface (Vanithakumari et al., 2013; Bartlet et al., 2018; Cao et al., 2018). On the other hand, the nanopillar structure on the superhydrophobic surface can kill the bacteria attached to the surface, but the mechanisms of microbial repulsion on superhydrophobic surfaces are complex and little understood currently. However, the characteristic of the hydrophobic surface repelling bacteria has certain limitations, most Gram-negative microorganisms exhibit repulsion, and Gram-positive microorganisms tend to adhere to these surfaces (Jaggessar et al., 2017). Table 3 lists a few examples of antibacterial superhydrophobic surfaces.

**TABLE 3:** Examples of bacteriostasis on superhydrophobic surfaces.

The name of the alloy	The preparation methods	Bacterial	The antibacterial effect	Reference
5,083 aluminum alloy	Ammonia etching and PFDTES modification	SRB	Greatly reduce the adhesion, growth, and proliferation of SRB.	Zhang et al. (2019)
Micro-nano structured titanium	Thermochemical treatment after silane modification	*S. aureus*	Decreased bacterial adhesion significantly (>90%) and prevented biofilm formation	Manivasagam et al. (2022)
		*E. coli*		
Flower-like micro-nano titanium particles	Electrophoretic deposition	*E. coli*	Repel *E. coli* adhesion	Zeng et al. (2020)
Aluminum	Passivation with low surface energy OTES molecules after chemical etching	*S. aureus*	An antibiofouling property of 99.9% against *S. aureus*, 99% against *P. aeruginosa* and 99% against *E. coli* bacteria	Agbe et al. (2020)
		*P. aeruginosa*		
		*E. coli*		
Superhydrophobic basalt scales (SiO_2_)	Fluorinated with PFDTES after NaOH solution chemical etching	*P. aeruginosa*	Inhibited the adhesion of the *P. aeruginosa* cells	Zheng et al. (2021)

PFDTES: 1H, 1H, 2H, and 2H-Perfluorodecyltriethoxysilane. OTES, octyltriethoxysilane; SRB, Sulfate-reducing bacteria.

The self-cleaning effect of superhydrophobic surfaces is attributable to their low surface energy, the structure of bacteria, and surface roughness. Suitable surface roughness reduces the contact area, and low surface energy coating limits adhesion (Kavitha Sri et al., 2020). *S. aureus* is more likely to adhere to titanium surfaces than *P. aeruginosa*, because spherical bacteria require lower surface energy to successfully adhere to titanium (Fadeeva et al., 2011). However, compared with that on smooth titanium surfaces, the adhesion of bacteria on the surface of superhydrophobic titanium nanoparticles after treatment is relatively reduced. This resistance to bacterial colonization may be due to the greatly reduced surface area required for bacterial adhesion (Zhan et al., 2021).

**FIGURE 2:**
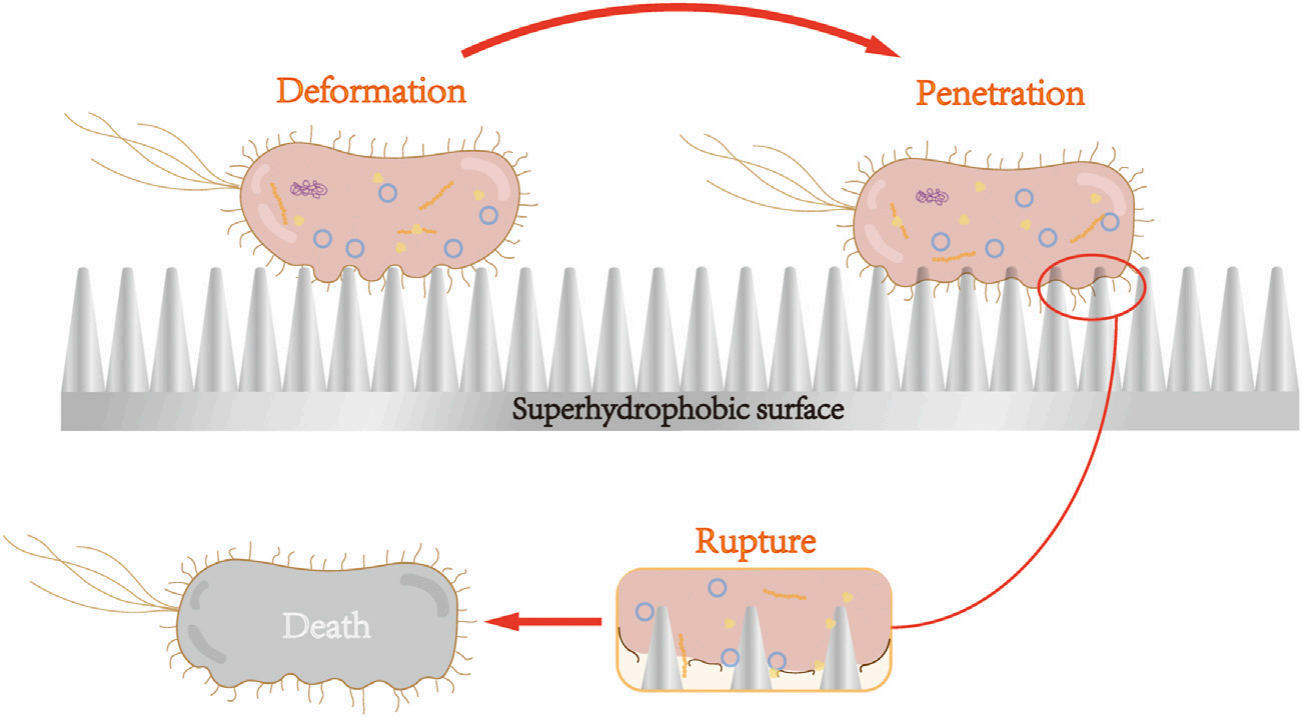
Nanoparticles of superhydrophobic surface cause bacterial cell membrane deformation, penetration, and rupture. After adhering to the surface of superhydrophobic nanoparticles, Gram-negative bacteria underwent cell membrane deformation, penetration, and rupture, and subsequent death.

Jenkins et al. (2020) reported that *Escherichia coli* adhering to the treated superhydrophobic nanoparticle titanium surface was first deformed under the action of nanoparticles, but the particles did not penetrate the cell membrane. Such deformation generally occurred in the area between the nanoparticles, namely air pockets, owing to the secretion of EPS layer. The bacterial cells then attached strongly to the nanostructure, and gradually, the nanoparticles penetrated the bacterial membrane. When the adhesion is sufficiently strong, the bacterial membrane ruptures owing to the resistance that occurs. Figure 2 shows the process of the bacterial rupture. By contrast, *S. aureus*, which clung to the surface, is not penetrated, likely because the cell walls of Gram-positive bacteria have thicker peptidoglycan layers, hindering the penetration of the nanoparticles.

Superhydrophobic surfaces can cause the attached Gram-negative bacteria to rupture and die *via* the surface morphology. Superhydrophilic surface is formed with antibacterial groups on the titanium surface through special surface treatment, which produces ROS to destroy bacterial cell membranes and cell walls, consequently leading to the death of bacteria.

## 4 Effect of superhydrophilicity titanium surface on osseointegration

The direct integration of bone and metal leads to structural and functional integration between the living bone and the implant surface, known as osseointegration, which is the rapid activation of the immune response to tissue injury *via* endosteal injury (Overmann et al., 2020). The osteoblast lineage is required for rapid osseointegration, and endothelial cell (EC)-mediated angiogenesis is required for new bone formation (Noble and Noble, 2014). Osseointegration can be divided into three stages: inflammation, repair, and remodeling (Sakka and Coulthard, 2009).

According to the sequence of the occurrence of osseointegration, we classified it into three parts as follows: immune response, angiogenesis, and osteogenesis. The effects of superhydrophilic surfaces will be introduced respectively.

### 4.1 Effect of superhydrophilicity titanium surface on immune response during osseointegration

The immune system is the most effective weapon against foreign body invasion and tissue damage. Osseointegration is actually an immune-driven process that relies on favorable inflammatory pathways that promote new bone formation as part of the host response to bioactive implants and reduce negative tissue responses that can lead to rejection. The primary driving force of bone immunology is host innate immunity, particularly macrophage activation (Lee and Bance, 2019). The immune cells that interact with the implant surface can release a variety of cytokines for regulating the microenvironment of the surrounding tissue, affecting the initial host response to the implants, the process of osseointegration, and the long-term effects of the implants (Zhang et al., 2021).

#### 4.1.1 Promotion of anti-inflammatory macrophage polarization through NETosis of neutrophils

After the degranulation of platelets (Terheyden et al., 2012), the neutrophils invade the blood clot via amoeboid migration, squeezing through little gaps in the walls of the blood vessels (Terheyden et al., 2012). Neutrophils can immediately dominate as the “first responders” after the tissue damage triggered by biomaterial implantation, and function in three primary abilities: the generation of oxidative bursts, release of granules, and formation of neutrophil extracellular traps (NETs), which enable neutrophil involvement in inflammation, recruitment of macrophages, M2 macrophage differentiation, resolution of inflammation, angiogenesis, and immune system activation (Selders et al., 2017).

Neutrophils dominate immediately after tissue injury (Wang, 2018). Although neutrophils exist for a considerably short time, they still play an indispensable role in promoting the polarization of macrophages. A role of neutrophils, NETosis, can be triggered in sterile inflammation (Thiam et al., 2020). It is a specific form of cell death caused by neutrophils, which is characterized by the release of cytokines, enzymes, immune cell recruitment chemokines, and DNA fibrils into the extracellular space referred to as NETs (Brinkmann et al., 2004; Yang et al., 2016). Abaricia et al. (2020) observed conditioned media from neutrophils grown on superhydrophilic titanium surfaces lead to anti-inflammatory macrophage polarization, and this anti-inflammatory effect was enhanced by the pre-treatment of neutrophils with a pharmacologic NETosis inhibitor. Therefore, a superhydrophilic titanium surface could reduce the neutrophil-induced pro-inflammatory transformation of macrophages regulated by NETosis.

#### 4.1.2 Regulation of macrophage polarization

The early inflammatory response of macrophages to the material surface prior to osteogenesis and angiogenesis determines the fate of the implant *in vivo* through bone immunoregulation (Bai et al., 2018). The effect of superhydrophilic surfaces on the immune system is predominantly reflected in promoting the polarization of macrophages to the anti-inflammatory phenotype (Bai et al., 2018; Gao et al., 2020; Huang et al., 2021). The surfaces induced the immune response of macrophages, which secreted initial levels of proinflammatory cytokines and ultimately the highest concentrations of anti-inflammatory and immunomodulatory factors (Hotchkiss et al., 2016). Anti-inflammatory factors, IL-4, IL-10, IL-13, and TGF-β, were upregulated, whereas the pro-inflammatory factors, IL-1, IL-6, and TNF-α, were significantly down-regulated. The superhydrophilic surface effectively inhibited the inflammation of the implant-bone interface via down-regulating the expression of iNOS and CD86 in the M1 phenotype and up-regulating the expressions of IL-10, CD163, and CD206 in the M2 phenotype (Bai et al., 2018).

TNF-α, a key proinflammatory regulator that is predominantly released by stimulated macrophages, enhanced osteoclast differentiation and resorption activity, inhibited osteoblast activity and bone formation (Theiss et al., 2005), also combined with NF-κB through NF-κB-TNF-α pathway to attenuate the macrophage immune response (Robson et al., 2004). The activation of the NF-κB pathway, a key intercellular regulator of inflammatory signaling, promotes the secretion of proinflammatory cytokines, including TNF-α and IL-1β (Dai et al., 2015).

**FIGURE 3:**
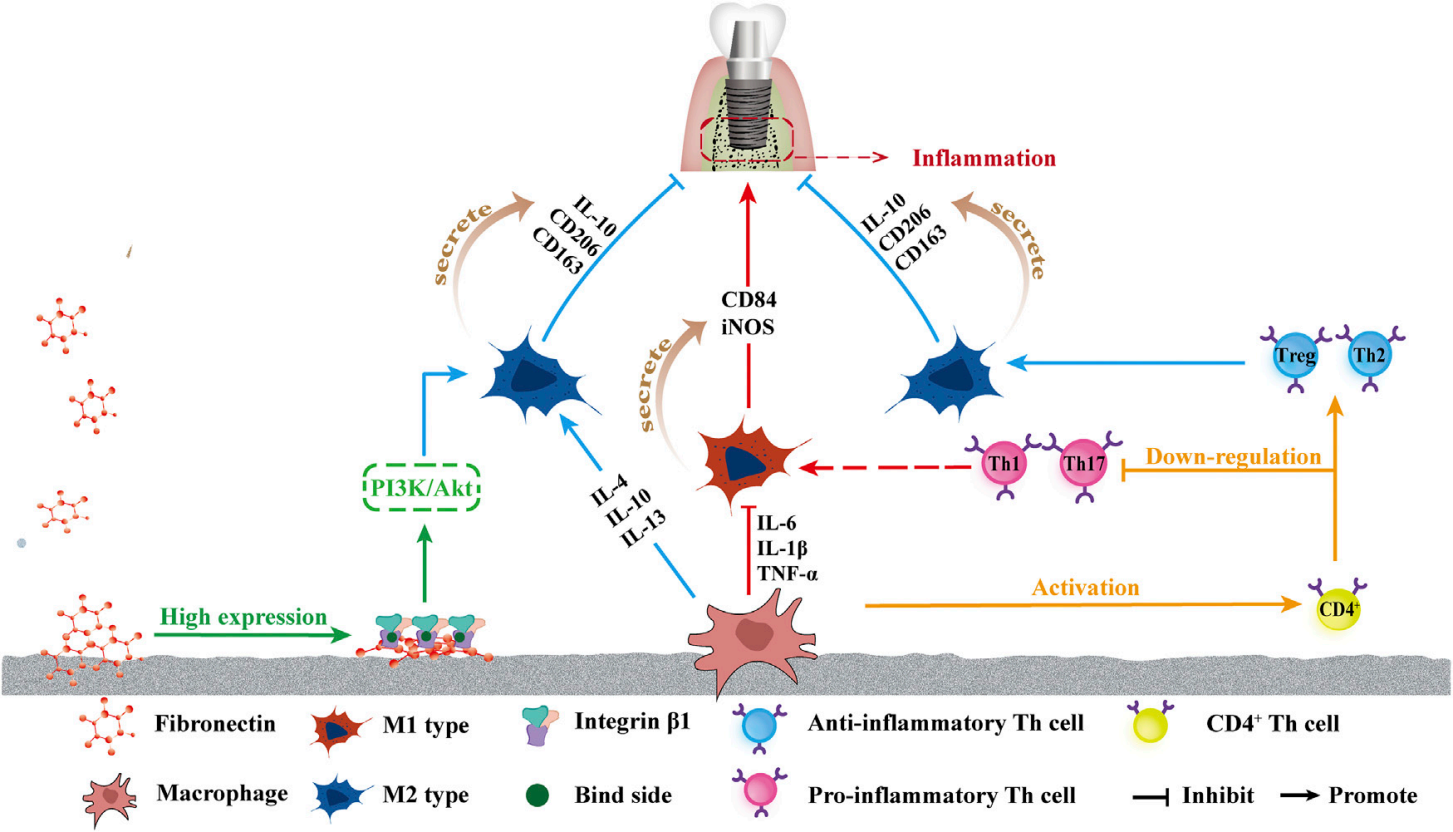
Schematic of influence of superhydrophilicity surface on osteogenic immune response. From left to right: the increased attachment of Fn provides more binding sites for integrin β1, which in turn promotes the polarization of macrophages towards M2 by promoting the PI3K/Akt pathway. The superhydrophilic surface up-regulates IL-4, IL-10, and IL-13, promotes the polarization into M2, and promotes M2 phenotype secretion of IL-10, CD206, and CD136, promoting anti-inflammatory effects. The down-regulation of IL-6, TNF-α, and IL-1β inhibited the polarization into M1 polarization, and inhibited the secretion of CD84 and iNOS of the M1 phenotype, inhibiting inflammation. Macrophages activated CD4^+^T cells, and the superhydrophilic surface promoted the differentiation of CD4^+^T cells into the anti-inflammatory phenotype Th2 and Treg and inhibited the differentiation into the pro-inflammatory phenotype Th1 and Th17.

In addition to the TNF-α-NF-κB pathway, integrin β1 has also been observed to contribute to osteogenesis via superhydrophilic surfaces in a study by lv et al. (2018) and the high expression of integrin β1 was detected on the UV-Ti surface, likely because fibronectin (Fn) maintains a more active conformation on the hydrophilic surface, leading to more cell binding sites (RGD) exposure, allowing integrin β1 to better bind to the hydrophilic surface (Li et al., 2020). The highly expressed integrin β1 is likely to drive macrophages to the M2 phenotype through the Phosphoinositol-3-kinase (PI3K)/Serine/threonine kinase (Petzold et al., 2017) signaling pathway. PI3K also signals through Akt to inhibit NF-κB activation, which induces a proinflammatory phenotype of macrophages, thereby inhibiting the polarization of macrophages toward the M1 phenotype (Lv et al., 2018). Hotchkiss et al. (2016) also reported that the combination of increased surface roughness and hydrophilicity may have a synergistic effect on increasing anti-inflammatory macrophage activation and yielding a suitable microenvironment, which may improve osseointegration and lead to a superior implant effect. Figure 3 shows the effects of a superhydrophilic surface on macrophage polarization.

#### 4.1.3 Promotion of the macrophage-induced adaptive immune response towards Th2 pro-wound healing phenotype

Newly recruited adaptive immune cells known as T cells are activated via antigen presentation by macrophages or dendritic cells (Lazarevic et al., 2013). Activated T cells, particularly CD4^+^ helper T cells, are considered the most influential cells for generating long-term immune responses. Helper T cell subsets have several phenotypes: helper cell type 1 (Th1), helper cell type 2 (Th2), helper cell type 17 (Th17), and T regulatory cells (Tregs). Th1 and Th17 are broadly considered proinflammatory (Lazarevic et al., 2013; Song et al., 2014), whereas Th2 and Treg helper cells are considered the most important for tissue regeneration (Lei et al., 2015; Schiaffino et al., 2017). In Hotchkiss’ *in-vivo* research, rough superhydrophilicity surfaces produced the maximum up-regulation of Th2 and Treg genes and down-regulation of Th1 and Th17 genes three days after implantation, demonstrating that M φ could polarize the adaptive immune response toward Th2, pro-wound healing phenotype, promoting the resolution of inflammation and increasing stem cell recruitment around implants (Hotchkiss et al., 2018), as shown in Figure 3.

### 4.2 Effect of superhydrophilicity titanium surface on angiogenesis

Newly formed capillaries play a critical role in this process and provide a favorable biological basis for implant osseointegration. The capillary system is the most basic structure to maintain the normal metabolism of the body, providing nutrients required for metabolism, exchange of oxygen and carbon dioxide, and a huge network of official channels for the exchange of the body and metabolites (Zhao et al., 2018).

An et al (2009) reported that superhydrophilic surfaces promote vascular EC proliferation by up-regulating related markers and expression factors, such as endothelial markers and angiogenic factors, Von Willebrand factor, thrombomodulin, and endothelial protein C receptors.

In the inflammatory stage, macrophages are stimulated by an intracellular transcription factor known as hypoxia-inducible factor (HIF-1), which may interact with VEGF to increase angiogenesis during osseointegration on the surface of superhydrophilicity implants (Calciolari et al., 2018; Zhang et al., 2020). The binding of VEGF-A to its receptor (VEGFR2) can activate various signaling pathways (El Chaar et al., 2019), leading to promoted cell survival, proliferation, infiltration, and migration (Lu et al., 2018). After the homodimerization of VEGF and VEGFR2, NO is stimulated, contributing to vascular permeability and long-term response of EC survival, migration, and proliferation (Rabelink and Luscher, 2006). Osteoblast-derived VEGF acts on adjacent ECs and stimulates osteoclast formation and differentiation (Hoeben et al., 2004; Liu et al., 2012; Hu and Olsen, 2016). Raines et al. (2019) *.* showed that superhydrophilicity titanium surfaces increased osteogenic VEGF-A expression. Upon binding of VEGF-A to VEGFR2, occurs homologous dimerizationand undergoes intense autophosphorylation, inducing downstream phosphorylation of PI-3 kinase in ECs (Maes et al., 2010; Calciolari et al., 2018; Raines et al., 2019).

### 4.3 Effect of superhydrophilic titanium surface on osteogenesis

Bones are continually adapted and remodeled by the activity of two cell types: mesenchymal stem cells that differentiate into osteoblasts, the immature cell-rich braided bone that forms through ossification, and osteoclasts that act on the resorption of bone derived from macrophage/monocyte lines (Sartori et al., 2019). The phenotypic differentiation of MSCs into osteoblasts is an important step in bone formation and implant integration (Kunrath et al., 2020). This process is regulated by the TGF-β\BMP2 signal, and TGF-β, as well as BMP2 expressions, are significantly increased on the superhydrophilic surface (Ivanovski et al., 2011). The surface interaction between titanium implants and osteoblastic membranes consists of two stages: the nonspecific interactions of membranes using electrostatic forces and environmental binding involving the entire assembly in local contact (Rahnamaee et al., 2020). Calciolari reported that specific signaling pathways, such as Wnt, VEGF, and mitogen-activated protein kinases (MAPK) at the genomic and proteome levels have been identified as modulated by differences in titanium surface hydrophilicity; additionally, the enhanced osteogenic response on the hydrophilic surface may be caused by the up-regulation of the PI3K/Akt signaling pathway (Calciolari et al., 2018).

#### 4.3.1 Superhydrophilicity surface with osteoblasts

Osteoblasts cultured on superhydrophilic surfaces showed a favorable diffusion performance, increased the contact area with materials, triggered osteogenic stimulation (da Silva et al., 2020), further promoted cell proliferation and differentiation (Li et al., 2019), and up-regulated related genes (Zhao et al., 2005). Studies have demonstrated that the osteoblasts cultured on the superhydrophilic titanium surface exhibited the enhancement of migration and proliferation ability (Henningsen et al., 2018; Smeets et al., 2019). Cold plasma treatment can promote the high expression of osteogenesis-related genes, such as alkaline phosphatase (ALP), Runt-related transcription factor 2 (Runx2), osteocalcin (OCN), and osteopontin (OPN) in precursor osteoblasts (Seo et al., 2014). An *in vivo* study by Tsujita et al, (2021) demonstrated that plasma-treated titanium could inhibit oxidative stress in cells and promote new bone formation around implants. In addition, Ann Wennerberg et al. (2014) compared the effects of the hydrophobic structure, hydrophobic structure of nano-structure, low-density nano-hydrophilic structure, and high-density nano-hydrophilic structure on the bone healing of adult rabbits via animal experiments *in vitro*, and concluded that the bone reaction was realized under the combination of wettability and the presence of nano-structure. The modified hydrophilic surface increased the absorption of plasma fibronectin (Rupp et al., 2004), promoted the differentiation of osteoblast cells, and upregulated related genes (Zhao et al., 2005).

The BMP-Runx2 pathway is a potential pathway that promotes osteogenesis on superhydrophilicity surfaces. Bone morphogenetic protein (BMP) is a member of the multifunctional cytokine transforming growth factor -β (TGF-β) superfamily and is an important factor for osteogenesis. After BMP binds to its receptor (BMPR), BMPR is recruited to form an activated quaternary complex, which subsequently phosphorylates and activates the intracellular Smad protein. The receptor Smad binds to co-Smad and is transported to the nucleus as a transcription factor. Runx2 is a key transcriptional regulator of osteoblast differentiation, and one of the BMP-Smad target genes is Runx2. Runx2 binds to the OCN promoter and is involved in the early expression of osteochondral progenitor cells and osteoblast differentiation. Runx2 also induces the expression of osteogenic markers such as OCN and OPN (Lin and Hankenson, 2011). Both BMP and Runx2 are highly expressed on superhydrophilic surfaces, indicating that the superhydrophilicity promotion of bone integration may be closely related to the BMP-Runx2 pathway.

Additionally, the forkhead box transcription factor O1 (FoxO1) involves the interaction between the superhydrophilic surface and osteoblasts. Huang reported that the hydrophilic surface can reduce the level of ROS in macrophages under oxidative stress, and promote the inflammatory response to the anti-inflammatory type by upregulating FoxO1 (Huang et al., 2021). FoxO1 mediated the antioxidant and osteo-differentiation effects. Previous studies have also demonstrated that the appropriate upregulation of FoxO1 activates transforming growth factor-β1 (TGF-β1), a key growth factor in wound repair, and protects the cells against oxidative stress (Ponugoti et al., 2013). Further molecular mechanism experiments showed that hydrophilic surfaces promoted FoxO1 expression under oxidative stress and also promoted osteogenic differentiation (Huang et al., 2021).

**FIGURE 4:**
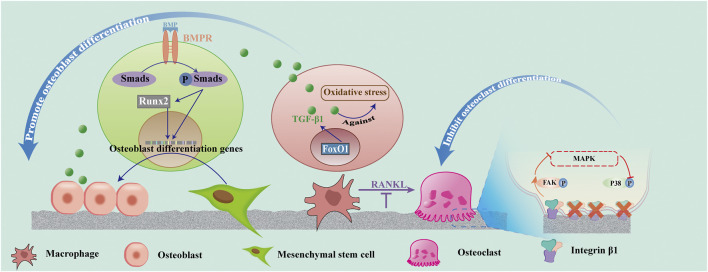
Effect of superhydrophilicity surface on osteogenic associated cells. Superhydrophilic surfaces promote osteogenic differentiation by promoting the activation of the BMP-Smad-Runx2 pathway in MSC, up-regulating FoxO1-TGF -β1 expression in macrophages, and promoting osteogenic differentiation. Osteoclast differentiation was suppressed by inhibiting RANKL and integrin β1/FAK/MAPK pathways.

In conclusion, superhydrophilic surfaces might up-regulate the high expression of BMP and Runx2 and promote FoxO1 gene expression to up-regulate TGF-β1, inhibit inflammation, as well as promote osteoblast proliferation and differentiation. Figure 4 shows the process of the BMP-Smad-Runx2 pathway and FoxO1-TGF-β1pathway. Thus, controlling the inflammation of the bone and surrounding tissues at an appropriate level is the key to promoting ideal osseointegration and reducing peri-implant bone resorption.

#### 4.3.2 Superhydrophilicity surface with osteoclasts

Osteoclasts, a key cell in the remodeling stage, can be formed via macrophage differentiation stimulated by the receptor activator nuclear factor-Kappa B ligand (RANKL) in the presence of at least three nuclei (He et al., 2022). ECs support vascular-associated osteoclast differentiation through RANKL-RANK signaling (Zhang et al., 2020).

The inflammatory mediators, such as IL-1, IL-6, and TNF-α, secreted by the M1 macrophages, can increase the level of RANKL, an important cytokine that regulates the formation of osteoclasts, induce the death of osteoblasts (Sun et al., 2021), and promote the activation of macrophages into osteoclasts (Insua et al., 2017). Osteoclasts appeared in the wound several days after surgery. They begin to create space for new bone formation and remove primary bone contact. The remodeling phase may continue for several years until most of the old bone in contact with the original bone is replaced by the newly formed, load-oriented bone (Terheyden et al., 2012). Studies have demonstrated that superhydrophilic surfaces increase macrophage recruitment and decrease osteoclast formation. However, the integrin β1 expression was decreased in osteoclasts on the nanotube surface compared with the untreated titanium surface. The surfaces of superhydrophilicity titanium nanotubes inhibited the differentiation of osteoclasts and promoted osteogenesis by decreasing integrin β1-mediated FAK phosphorylation and its downstream MAPK pathway (P-P38). Moreover, the activity of osteoclasts on the nanotube surfaces was decreased (He et al., 2022).

## 5 Conclusion and perspectives

Owing to the unfavorable osseointegration and implant failure caused by microbial-related infections in clinical practice, implant materials that combine antibacterial properties and biocompatibility have always been an important goal for obtaining the perfect initial implantation effect and for maintaining the long-term survival of implants. Among the various surface modification methods, improving the wettability of the implant surfaces has been considered to regulate the host response to the implants, thereby accelerating the osseointegration speed; the superhydrophilic surfaces can possess the above functionality as well as show certain antibacterial effects. In this study, advances in superhydrophilicity titanium alloys, including antibacterial function and improved biocompatibility, are reviewed, and the related mechanisms in recent research are summarized. The post-treated titanium surfaces usually perform their antibacterial function by inhibiting bacteria adhesion and cell viability and even partially eliminating bacteria. Moreover, because of the favorable biocompatibility, a superhydrophilic titanium surface could effectively modulate the macrophages with an enhanced immune response against bacteria and influence the race between macrophages and bacteria to adhere to biomaterial surfaces (Yang et al., 2021). Thus, the superhydrophilic surface considerably reduces the likelihood of failure of the implant to bond to the bone surface owing to microbial infection. It promotes osteogenic immune responses as well as angiogenesis and osteogenic differentiation.

However, numerous challenges remain to be overcome. First, the mechanisms of the obtained antibacterial properties, based on the treatment methods, require further investigation. Current studies have observed that the superhydrophilicity of titanium treated with UV or cold plasma could inhibit bacterial adhesion and proliferation in a time-dependent manner which commonly lasts for over 24 h, longer than the 6-h decisive period post-implantation. The different durations of the surface antibacterial properties are related to multiple factors, including the treatment methods, bacterial species, and inherent composition of the biomaterials. Furthermore, the duration of the superhydrophilic surface treatment could influence the antibacterial effect, but the effect has certain limitations compared with other antibacterial methods. Therefore, it is of significance to combine superhydrophilicity treatment with other surface modifications to exert better antibacterial properties while obtaining superior biocompatibility. Secondly, the specific effects of surface chemical composition changes on osteoblast-related cells need to be further studied. It has been found that the decrease of carbon content on the titanium surface is beneficial to improve the biological activity, and the increase of oxygen content is beneficial to increase the oxygenated fraction that can absorb fibronectin, and improve the protein adsorption rate to regulate the proteoglycan and cytoskeleton structure. Therefore, the effect of chemical composition changes on osteogenesis is worthy of further study. Thirdly, more *in vivo* studies are required, particularly to assess its effect on long-term implantation, which will be key to long-term clinical use. The biocompatibility and mechanical strength of the coating, such as the mechanical stability of the superhydrophilicity surface of titanium alloy, whether the propagation of the biological coating can withstand the biological environment of the human body, and whether the exposure to metal oxides will interfere with the function of cells and organs, have not been confirmed. Therefore, it is important to determine the stability and cytotoxic behavior of this material/implant. Fourthly, the durability of the structure has not been proven, and despite the significant efforts made to date, achieving superhydrophilic surfaces with high mechanical strength, favorable chemical stability, and durability to meet demanding applications remains a challenge, and further research is required. For numerous applications that do not require wear resistance, superhydrophilic surfaces can be used for a favorable performance. For example, superhydrophilic surfaces can be applied to permanent implants, reduce bacterial adhesion, and promote implant bone integration. Therefore, it is important to understand the durability requirements of the target application to adopt the appropriate treatment methods when preparing suitable superhydrophilic surfaces. Finally, the artificial structure is far from emulating the natural structure. The superhydrophilicity of natural structures and other properties conferred by them cannot be fully reflected in artificial surfaces.

In conclusion, while the superhydrophilicity obtained by treating titanium alloys alone holds considerable promise for the development of next-generation orthopedic and dental implants, more work and sustained effort are required to translate them into devices for clinical applications. In addition, determining methods to prove the sustainable superhydrophilicity of the material surface in the body after implantation remains a challenge to be overcome at present.
